# Immunosuppressive regimens for adult liver transplant recipients in real-life practice: consensus recommendations from an Italian Working Group

**DOI:** 10.1007/s12072-020-10091-5

**Published:** 2020-10-24

**Authors:** Umberto Cillo, Luciano De Carlis, Massimo Del Gaudio, Paolo De Simone, Stefano Fagiuoli, Francesco Lupo, Giuseppe Tisone, Riccardo Volpes

**Affiliations:** 1 Hepatobiliary and Liver Transplant Unit, University Hospital of Padua, Padua, Italy; 2 Department of General Surgery and Transplantation, Niguarda Hospital, Milan, Italy; 3 School of Medicine, University of Milano-Bicocca, Milan, Italy; 4 Department of General Surgery and Transplantation, Policlinico S. Orsola-Malpighi, Bologna, Italy; 5 Hepatobiliary Surgery and Liver Transplantation Unit, University of Pisa Medical School Hospital, Pisa, Italy; 6 Gastroenterology Hepatology and Transplantation, Papa Giovanni XXIII Hospital, Piazza OMS, 124127 Bergamo, Italy; 7 Department of General Surgery, Azienda Ospedaliera Città Della Salute E Della Scienza, Turin, Italy; 8 Department of Experimental Medicine and Surgery, Transplantation Surgery, Policlinico Tor Vergata University of Rome, Rome, Italy; 9 Mediterranean Institute for Transplantation and Advanced Specialized Therapies (ISMETT/IRCCS), Palermo, Italy

**Keywords:** Calcineurin inhibitor, Chronic kidney disease, Consensus recommendations, Everolimus, Hepatocellular carcinoma, Immunosuppression, mTOR inhibitor, Sirolimus, Solid-organ transplantation, Tacrolimus

## Abstract

It is a well-recognized fact that implementing new guidelines in clinical practice may be difficult; therefore the Italian Society for Organ and Tissue Transplantation (SITO) set out to define practical immunosuppression tools for the management of liver transplantation patients. In 2017, an Italian Working Group of liver transplant experts and hepatologists issued a set of consensus statements along with evidence-based recommendations on the use of everolimus after liver transplantation. This article presents the evidence- and consensus-based algorithms developed within the Italian Working Group, which are aimed towards guiding clinicians in the selection of immunosuppressive regimens for the management of adult liver transplant recipients in real-life practice. The liver transplant recipient population, typically managed in clinical practice, was divided into the following categories: (1) standard patients; (2) critically ill patients; (3) patients with a specific etiology; (4) patients with hepatocellular carcinoma; (5) and patients with de novo malignancies. The algorithms are divided into two parts, according to the time from transplantation (0–3 months and > 3 months) and are discussed here along with relevant supporting literature, when available. Ultimately, it is hoped that the evidence- and consensus-based algorithms developed within the Italian Working Group, and presented here, contribute to simplify, personalize, and optimize immunosuppression of liver transplantation recipients in clinical practice.

## Introduction

Despite considerable progress in solid organ transplantation leading to increased patient and graft survival, complications associated with maintenance immunosuppressive therapy required to prevent rejection remain a major issue [1]. In liver transplant recipients, for example, the chronic use of calcineurin inhibitors (CNIs) has been shown to increase the risk of renal dysfunction, metabolic disorders, neurotoxicity, and de novo malignancies [2–8]. As an example, approximately 20% of liver transplant recipients experience chronic renal failure within 5 years of transplantation [7].

Consequently, extensive efforts have been devoted to developing strategies for reducing or withdrawing CNIs [9]. In this regard, the mTOR inhibitors, sirolimus, and everolimus have attracted considerable attention because of their immunosuppressive and antiproliferative properties [1]. Furthermore, a large body of evidence has shown that mTOR inhibitor-facilitated CNI reduction is associated with nephroprotective effects in both de novo and maintenance liver transplant recipients [10–14].

In 2017, an Italian Working Group, composed of senior representatives from Italian liver transplant centers, issued a set of consensus statements with evidence-based recommendations on the use of everolimus after liver transplantation [15]. The statements addressed four areas of practical interest: (1) kidney function; (2) timing of everolimus introduction, reduction of CNI exposure, and risk of graft rejection; (3) antiproliferative effect of everolimus; and (4) management of everolimus-related adverse events [15]. According to these recommendations, strategies for the prevention of renal impairment should be implemented early following transplantation to obtain good outcomes (typically after 4 weeks); in addition, owing to its antiproliferative properties, everolimus is also recommended for the prevention of de novo malignancies and recurrence of hepatocellular carcinoma (HCC) [15].

However, the implementation of new recommendations is rarely a straight-forward process in real-life. Indeed, the multicenter, observational study SURF, which assessed the management of patients undergoing liver transplantation in Italy, has shown that most liver transplant recipients with chronic kidney disease are not managed according to current guidelines, with less than 20% being switched from standard CNI-based immunosuppressive regimens to renal-sparing alternatives [16]. In addition to the previously mentioned recommendations [15], a further objective of the Italian Working Group of transplantation experts was to identify practical issues not fully covered by the guidelines and to improve the implementation of these recommendations in clinical practice.

This article presents the evidence- and consensus-based algorithms developed within the Italian Working Group and aimed at guiding clinicians in the selection of immunosuppressive regimens for the management of various categories of adult liver transplant recipients.

## Methods

This project was conceived under the auspices of the Italian Society for Organ and Tissue Transplantation (SITO) to define practical immunosuppression algorithms for the management of adult, ABO-compatible liver transplantation patients and was prompted by the awareness that implementing new guidelines in clinical practice may be difficult. It was launched in 2017 by a group of 8 Italian expert transplant physicians (scientific board), most of whom were among the authors of the recommendations for the use of everolimus in liver transplantation published in the same year in Transplantation [15]. The expertise of the scientific board members was substantiated by their publication records, participation in national/international scientific meetings and clinical trials, academic rank, and clinical experience in liver transplant surgery or transplant hepatology, as well as the use of mTOR inhibitors. With regard to everolimus use, it should be noted that in Italy it has been used off-label for liver transplantation since 2006, well before the EMA approval in this indication, thanks to specific legislation and based on evidence from phase II clinical trials. The aim of this project was to produce evidence- and consensus-based recommendations for immunosuppressive therapy in adult liver transplant recipients, with a focus on clinical practice.

In 2017, the scientific board convened in Milan to share and discuss the clinical experience with everolimus at their centers and to review the relevant literature, with the purpose of identifying unresolved issues and producing a standard protocol for immunosuppression, in line with current recommendations for everolimus use [15] and based on published evidence when available, clinical experience, and consensus. The scientific board used a three-step modified Delphi method for reaching consensus, as previously described [15].

In Step 1, the scientific board convened in Milan at a face-to-face meeting during which it was decided to divide the liver transplant recipient population, typically managed in clinical practice, in the following categories: (1) standard patients; (2) critically ill patients; (3) patients with a specific etiology; (4) patients with HCC; (5) and patients with de novo malignancies. ABO-incompatible liver transplant patients were excluded from the target population due to the scarcity of such practice in Italy. With respect to HCC patients, previous national consensus conferences suggested liver transplantation for T2 patients (≥ 2 cm), whilst the upper tumor size limit or stage was left to the discretion of each transplant center based on donor graft availability and transplant benefit [17]. The scientific board drafted immunosuppressive protocols for each patient category. These protocols were to be submitted for assessment to a panel of clinicians (consensus panel). The consensus panel was composed of clinicians from Italian liver transplant centers (21 centers were invited; 16 centers participated with a total of 31 representatives; names and affiliations of clinicians are listed in Appendix A).

In Step 2, the scientific board and consensus panel met in Rome at a plenary meeting. The members of the consensus panel were asked to express their agreement or disagreement on the treatment protocol proposed for each patient category using a Likert scale from 1 to 5 (1, strongly disagree; 2, disagree; 3, neutral; 4 agree; 5 strongly agree). The positive consensus was defined by > 70% agreement on a given protocol (expressed as the percentage of respondents scoring the protocol with either 4 or 5) [15]. A positive agreement was reached on all immunosuppression protocols.

The treatment algorithms presented here were finalized during a final face-to-face meeting of the scientific board held in Milan (Step 3). The algorithms have been available since September 2018 on the SITO website (https://www.societaitalianatrapiantidiorgano.com/).

After a period of real-world practice, the treatment algorithms were amended and approved by the consensus panel at a plenary meeting held in December 2019.

## Strategies for immunosuppression in liver transplant recipients

**Fig. 1:**
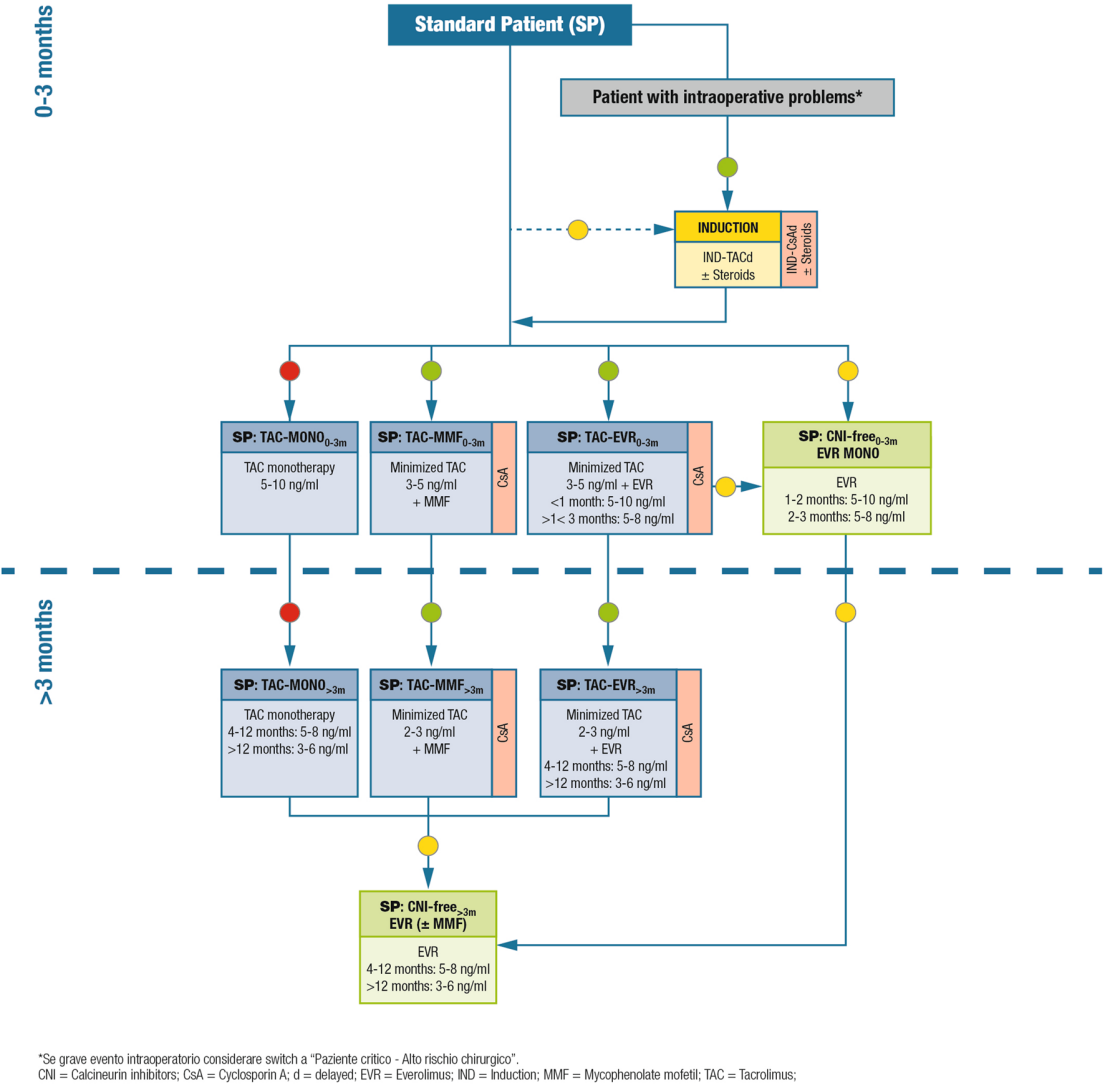
Immunosuppression algorithm for standard patients. Key: green circle = recommended; yellow circle = caution advised; red circle = not recommended

**Fig. 2:**
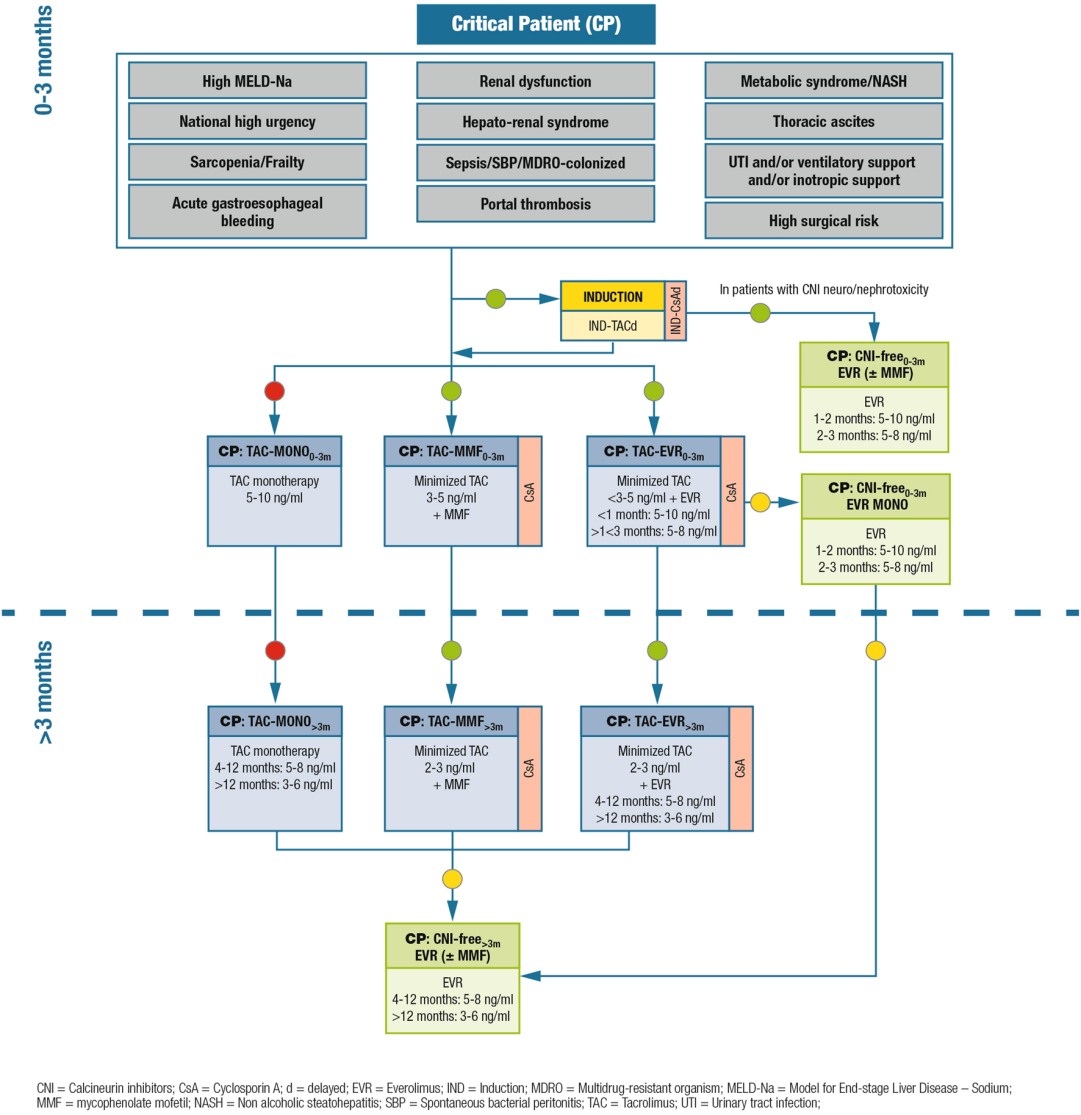
Immunosuppression algorithm for critically ill patients. Key: green circle = recommended; yellow circle = caution advised; red circle = not recommended

**Fig. 3:**
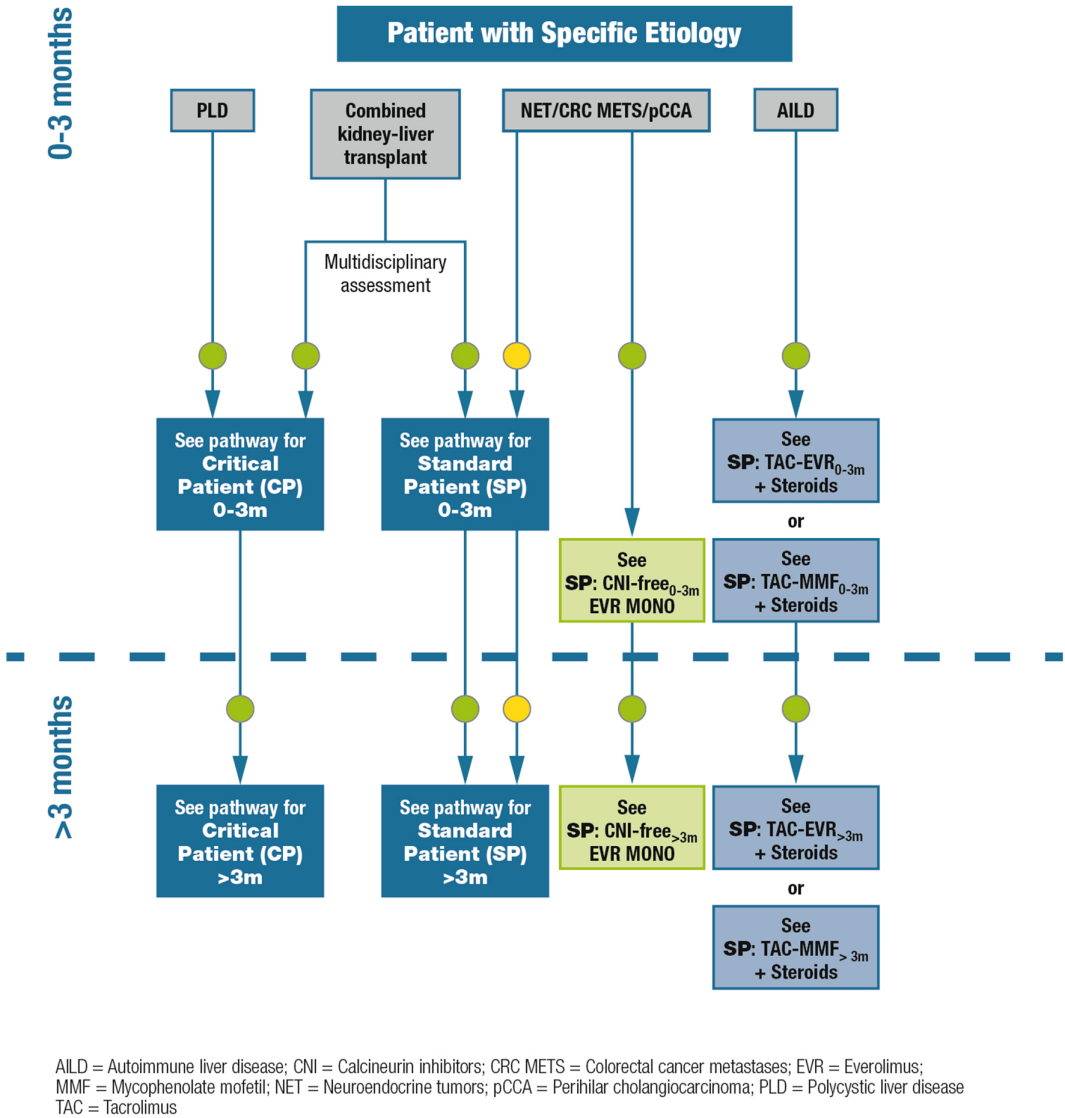
Immunosuppression algorithm for patients with a specific etiology. Key: green circle = recommended; yellow circle = caution advised

**Fig. 4:**
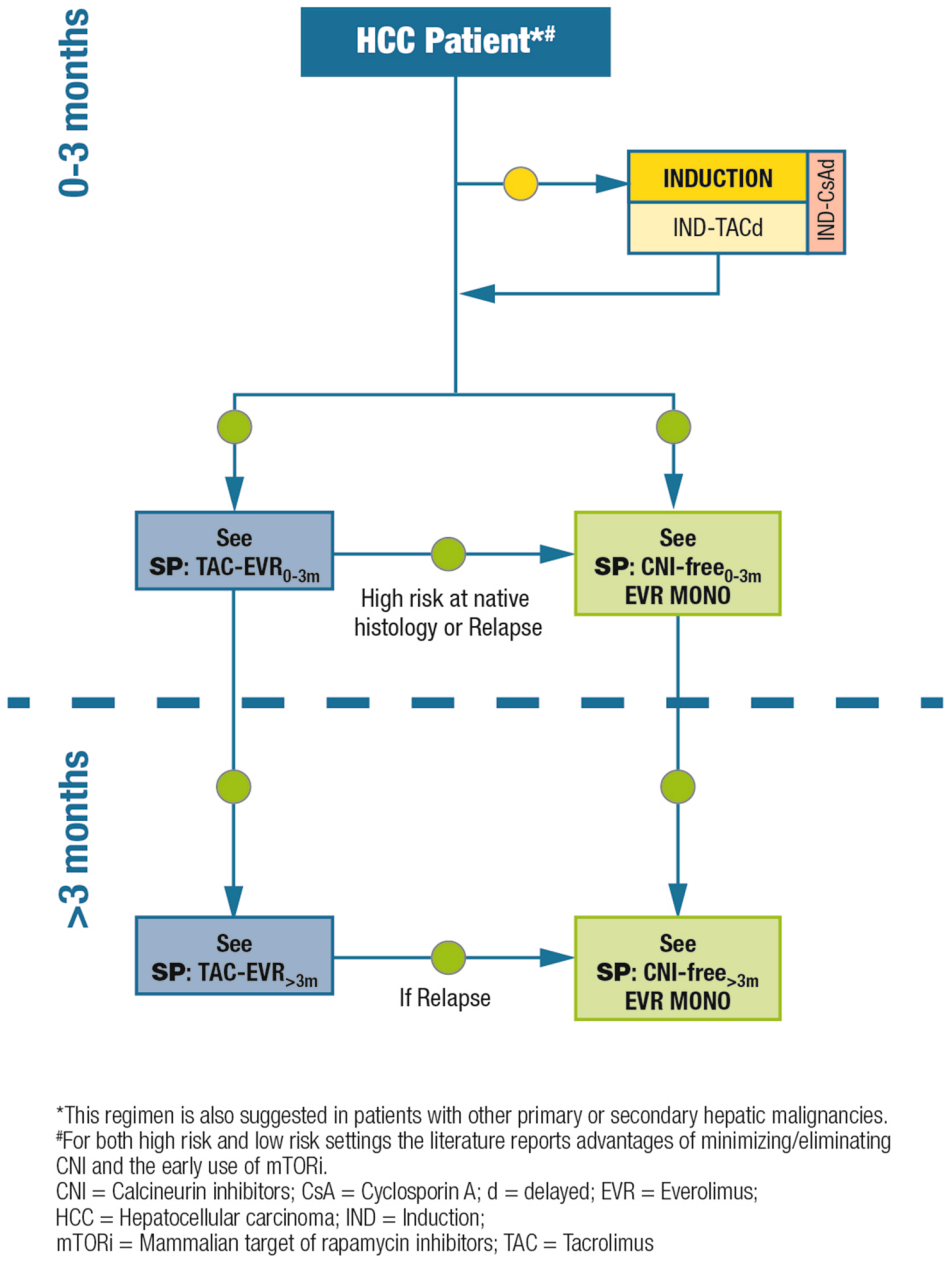
Immunosuppression algorithm for patients with hepatocellular carcinoma. Key: green circle = recommended; yellow circle = caution advised

**Fig. 5:**
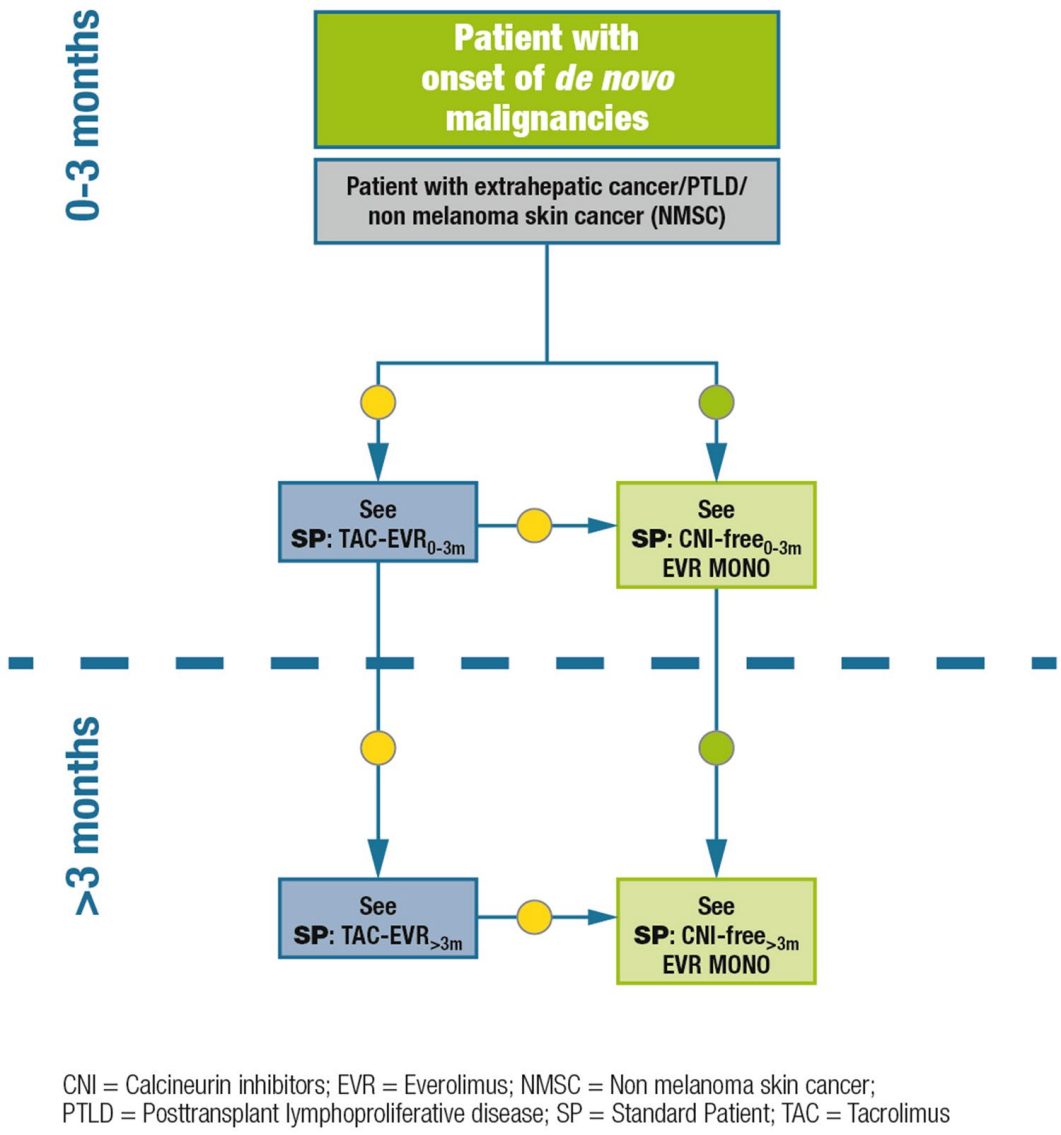
Immunosuppression algorithm for patients with de novo malignancies. Key: green circle = recommended; yellow circle = caution advised

The algorithms for immunosuppressive therapy in adult, ABO-compatible liver transplant recipients, according to the patient category, are shown in Figs. 1–5. All algorithms are divided into two parts, according to the time from transplantation: 0–3 months and > 3 months. General remarks on the algorithms are shown in Table 1 and additional comments concerning the different recommended immunosuppressive therapies are shown in Table 2. The algorithms are discussed in the following sections along with relevant supporting literature, when available.

**Table 1:** General remarks on the algorithms for immunosuppressive therapy in liver transplant recipients

	General remarks
Blood levels	The indicated target values are not binding
Steroids	The orientation of the algorithm is steroid-free
CNI	As an alternative to tacrolimus, cyclosporine can be chosen (in particular in dysmetabolic patients with severe diabetes)
Induction	ATG can be chosen as an alternative to anti-IL-2R
CNI-free schemes in which MMF is associated with everolimus	Evaluate a reduction of everolimus levels
MMF	As an alternative, the sodium salt form of MPA can be used

*ATG* anti-thymocyte globulin, *CNI* calcineurin inhibitor, *IL* interleukin, *MMF* mycophenolate mofetil, *MPA* mycophenolic acid

**Table 2:** Notes on the algorithms for immunosuppressive therapy in liver transplant recipients

Induction for different CNIs (IND-TACd / IND-CsAd)
Induction therapy is recommended to postpone the introduction of CNI to 3–5 days post-transplant
Dosages
• Anti-IL-2R (basiliximab) 20 mg on day 0 (within 6 h of reperfusion) + 20 mg to day 4 (p.o.)
• Tacrolimus 0.03–0.075 mg/kg/day between day 3 and day 5
• Cyclosporine 5 mg/kg every 12 h between day 3 and day 5
Target blood levels
• Tacrolimus 3–5 ng/mL
• Cyclosporine
3–5 days: 200–250 ng/mL
0–3 months: 150–200 ng/mL
> 3 months: 120 ng/mL
Steroids
Steroid-free orientation
If steroids
Dosage: bolus e.v. intraoperative 500–1000 mg
Tapering and interruption, ideally within 1 month, except for patients with AI diseases
Everolimus
Entry criteria
< 1 month: NO proteinuria > 1 g and PLT > 50,000
From 1 month to > 3 months NO if:
• Discards in the 2 weeks pre-therapy
• PLT > 50,000; leukocytes > 2500; Hb ≥ 8 and/or
• Hypertriglyceridemia > 250 mg/dL; hypercholesterolemia > 250 mg/dL and/or
• Proteinuria > 1 g; persistent ascites; wound infections; interstitial pneumonia
Mycophenolate mofetil
Entry criteria
• NO pancytopenia
• HCT > 26%
• PLT > 50,000 (+ 10,000)

*CNIs* calcineurin inhibitors, *IL* interleukin, *h* hours, *HCT* haematocrit, *Hb* hemoglobin, *PLT* platelets, *p.o.* by mouth

### Standard patients

#### Definition/description of patient category

Low-risk patients with a Model for End-Stage Liver Disease (MELD) score < 25 and no autoimmune disease, HCC, or history of renal dysfunction.

#### Relevant supporting evidence from the literature

Early (30 ± 5 days post-transplant) or very early (≤ 10 days post-transplant) mTOR inhibitor introduction to reduce exposure to CNI has been reported to improve estimated glomerular filtration rate (eGFR) by 8–12 mL/min/1.73 m^2^ at 1 year after transplantation [15]. In the H2304 study, which investigated de novo use of everolimus in 719 liver transplant recipients who were randomized at 30 ± 5 days post-transplant, the adjusted change in eGFR at 12 months was superior in the everolimus plus reduced tacrolimus group using the Modification of Diet in Renal Disease (four-variable) (MDRD4) formula (difference of 8.5 mL/min/1.73 m^2^; *p* < 0.001) [11], and remained significantly superior at 24 months (difference of 6.7 mL/min/1.73 m^2^; *p* = 0.002) [12] compared with the standard tacrolimus-based regimen. An extension of the H2304 study demonstrated significantly improved renal function preservation for up to 3-years post-transplantation in 282 liver transplant recipients receiving everolimus plus reduced tacrolimus, with mean eGFR decreasing from randomization to month 36 by 7.0 ± 31.3 mL/min/1.73 m^2^ versus 15.5 ± 22.7 mL/min/1.73 m^2^ in the tacrolimus-control group (*p* = 0.005) [18].

In the PROTECT trial, in which 203 liver transplant patients were randomized at 4 weeks post-transplant to discontinue CNI therapy and start everolimus or to continue their current CNI-based regimen, an eGFR treatment difference of 7.8 mL/min (*p* = 0.021) in favor of everolimus was identified at 12 months after transplantation using the MDRD4 formula [19]. At month 35 after randomization, a 10.1 mL/min benefit in the adjusted mean eGFR in favor of everolimus was identified using the Cockcroft-Gault formula (*p* = 0.082 vs. CNI), 9.4 mL/min/1.73 m^2^ (*p* = 0.053) using the MDRD4 formula, and 9.5 mL/min/1.73 m^2^ (*p* = 0.028) using the Nankivell formula [14].

A separate study, in which 78 liver transplant patients were randomized to early CNI withdrawal followed by everolimus as monotherapy (with the initial dose of everolimus started on day 10 after liver transplantation) or to cyclosporine, identified a significant improvement in renal function at 12 months after transplantation in patients treated with everolimus compared with cyclosporine (mean eGFR values [MDRD formula] were 87.6 ± 26.1 mL/min vs. 59.9 ± 12.6 mL/min, respectively; *p* < 0.001) [20].

In addition, evidence from randomized studies and retrospective analyses shows that the improvement in renal function is limited if CNI-reducing strategies are postponed until renal function has deteriorated (eGFR < 60 mL/min/1.73 m^2^) [10, 21–23].

#### Summary of recommendations and expert opinion

In standard patients (Fig. 1), immunosuppression is based on CNIs, usually tacrolimus; cyclosporine can also be used. Tacrolimus monotherapy is not recommended. Early CNI reduction is feasible with the introduction of everolimus or mycophenolic acid derivatives, and may be further facilitated by the administration of induction agents (usually basiliximab for liver transplant recipients). Overall, a steroid-free approach is recommended.

### Critically ill patients

#### Definition/description of patient category

Patients with one or more of the conditions listed in Fig. 2, namely high MELD-Na (i.e., MELD-Na > 29 or MELD-Na 25–29 with concomitant renal dysfunction/dialysis or chronic encephalopathy), national high urgency, acute gastroesophageal bleeding, renal dysfunction/dialysis, hepato-renal syndrome, sepsis, spontaneous bacterial peritonitis, colonization by multidrug-resistant organisms, portal thrombosis, metabolic syndrome or non-alcoholic steatohepatitis, thoracic ascites, urinary tract infection and/or ventilatory support and/or inotropic support, or high surgical risk are considered as high-risk patients. Frail patients, with sarcopenia as a distinctive characteristic, also belong to this category [24–27].

#### Relevant supporting evidence from the literature

Studies in this patient category are lacking, as most trials enroll low-risk patients (e.g., patients with no renal impairment).

#### Summary of recommendations and expert opinion

Induction therapy (basiliximab) is recommended in these patients to delay the introduction of CNI to 3–5 days post-transplantation. In patients with elevated MELD-Na (> 29), the use of induction therapy should be carefully considered; CNI should be initiated at lower doses as compared to standard patients. CNI monotherapy is not recommended, and cyclosporine is a possible alternative to tacrolimus in particular in patients with dysmetabolic abnormalities and decompensated diabetes. For CNI reduction, the available options include mycophenolic acid derivatives and everolimus. CNI discontinuation can be considered for patients with signs of CNI-induced neuro-nephrotoxicity. Everolimus is particularly indicated in patients at risk of renal dysfunction or with renal dysfunction confirmed by eGFR assessments; among these patients, those with diabetes or metabolic disease may particularly benefit from everolimus, as this mTOR inhibitor has cardiovascular protective effects beside nephroprotective properties. Hyperlipidemia and hypertriglyceridemia, if present, can be treated before or after the introduction of everolimus. Proteinuria requires accurate renal function work-up and can be treated with appropriate strategies.

### Patients with a specific etiology

#### Definition/description of patient category

In this group, we included less frequent or emerging indications to liver transplantation in Italy, as follows: patients with polycystic liver disease (PLD) with or without kidney involvement, patients necessitating combined liver-kidney transplantation, patients with autoimmune liver disease (AILD), patients with oncological indications to transplantation as neuroendocrine tumors (NET), metastatic colorectal cancer (metaCRC), or patients with cholangiocarcinoma (CCA) (Fig. 3).

#### Relevant supporting evidence from the literature

Two inherited disorders associated with PLD can be distinguished: isolated polycystic liver disease (PCLD) and autosomal dominant polycystic kidney disease (ADPKD). Although PCLD is not associated with excess mortality when left untreated, the quality of life supports the choice of liver transplantation as a therapeutic option [28, 29].

Combined liver-kidney transplantation should be considered in patients with severe renal impairment or on renal replacement therapy [30, 31].

Liver transplantation for NET liver metastasis may be a viable treatment option in selected patients as 5-year post-transplant overall survival rates of 47–97% and disease-free survival rates of 32–87% have been reported [32–34]. Survival outcomes after liver transplantation are similar for liver transplantation performed in “ideal” candidates and for those with HCC [33]. However, as recurrence rates remain higher, immunosuppressive treatment must be appropriate to the oncological status.

For patients with metaCRC, the role of liver transplantation requires further elucidation and definition by ongoing clinical trials and real-life practice [35]. Nonetheless, a Norwegian study of 21 patients with nonresectable colorectal liver metastases demonstrated a 5-year survival rate of 60% after liver transplantation, exceeding outcomes reported for chemotherapy [36]. In colorectal cancer patients with nonresectable liver-only metastases, the 5-year overall survival rate was 83% after liver transplantation, highlighting the importance of improved patient selection criteria for optimal survival rates [37].

CCA, the second most common liver cancer, is associated with a very poor prognosis [38]. Liver transplantation protocols for CCA show low survival rates, given that tumor size (> 2 cm) and multi-nodularity are risk factors for tumor recurrence and worse outcomes when compared with similar HCCs [39]. Nevertheless, clinically acceptable results were obtained in selected patients who underwent neoadjuvant chemoradiotherapy followed by liver transplantation [40]. In such cases, the immunosuppressive strategy must balance the risk of recurrence. There is also some experimental and clinical evidence on the antiproliferative effect of mTOR inhibitors in CCA-tumor cells [41] and in clinical studies [42].

#### Summary of recommendations and expert opinion

Patients with isolated PCLD can be referred to the critical patient path, due to complex surgery. Patients with ADPKD and renal impairment and/or in renal replacement therapy are considered for simultaneous or delayed combined liver-kidney transplantation [43, 44]. In this case, the immunosuppressive therapy is directed towards the critical patient path or towards the standard case path depending on the clinical conditions and after a careful multidisciplinary evaluation [30, 31].

Everolimus-facilitated CNI reduction should be implemented as early as possible, to avoid further damage to the kidney. In patients with oncological issues, the early introduction of mTOR inhibitors should be considered, and their use is also recommended in the case of post-transplant recurrence [45]. This could apply also to patients with additional risk factors for post-transplant de novo malignancy development (i.e. alcohol-related liver disease).

AILD should be treated with tacrolimus and corticosteroids, adjusting the latter based on efficacy and side effects; everolimus or mycophenolates can also be added. Cyclosporine can be used instead of tacrolimus [46], mostly in case of relapse.

### Patients with hepatocellular carcinoma

#### Definition/description of patient category

Patients undergoing liver transplantation due to HCC.

#### Relevant supporting evidence from the literature

Antiproliferative properties of mTOR inhibitors and data from the literature support the use of this drug class in HCC patients. A large body of preclinical and clinical evidence has shown that mTOR inhibitors can limit HCC recurrence and progression in liver transplant recipients [47–49]. Sirolimus-based immunosuppression was beneficial after liver transplantation with significantly higher patient survival post-transplantation for HCC in a multivariate analysis of 2491 patients (hazard ratio 0.53, 95% confidence interval [CI] 0.31–0.92; *p* ≤ 0.05) [48]. mTOR inhibitors were also associated with significantly lower rates of HCC recurrence after liver transplantation when compared with CNIs (8% vs. 13.8%; *p* < 0.001) in a systematic review of 3666 HCC liver transplant recipients [47]. However, significant benefits of mTOR inhibitors were only observed in patients within Milan criteria (i.e., low-risk patients) in a prospective, randomized, phase 3 clinical trial of sirolimus-based immunosuppression in 525 HCC liver transplant recipients [50]. Moreover, HCC recurrence-free and overall survival benefits of mTOR inhibitors were apparent for up to 3–5 years post-transplantation, but were not sustained thereafter. An analysis of the US Scientific Registry of Transplant Recipients suggested beneficial, albeit not statistically significant, outcomes in terms of lower rates of HCC recurrence and cancer-specific mortality in recipients of liver transplantations for HCC receiving sirolimus-based immunosuppression, however, effects appeared to be modified by patient age at transplantation, with better outcomes identified for transplant recipients > 55 years of age than those ≤ 55 years [51].

For patients with recurrent HCC post-transplantation, evidence from retrospective analyses and case reports suggests that mTOR inhibitors should be introduced to slow disease progression due to their antiproliferative activity [52–54]. The addition of everolimus to sorafenib in a 46-year old male who experienced HCC recurrence 11 years after liver transplantation led to an approximately 50% reduction in tumor size after 3-months of treatment, with further reductions of tumor size after 8 months [52]. In a retrospective cohort study of 31 patients with recurrent HCC after liver transplantation, the combined use of mTOR inhibitor (everolimus or sirolimus) and sorafenib identified a median overall survival of 19.3 months after initiation of the combined treatment, with a median time to disease progression of 6.77 months [54]. In a retrospective analysis of 7 liver transplantation recipients with HCC recurrence who were switched to everolimus plus sorafenib, 5 patients were alive after a median follow-up of 6.5 months (interquartile range [IQR] 14 months), 4 patients with tumor progression; the median time to progression was 3.5 (IQR 12) months [53].

Compared with CNI-based immunosuppression, mTOR-inhibitor-based immunosuppression significantly increased recurrence-free-survival at both 1- and 3-years post-transplantation (risk ratio [RR] 1.09 and 1.1, respectively) in a systematic review and meta-analysis of 23 studies on the survival and recurrence of HCC in liver transplant recipients [55]. In addition, treatment with mTOR inhibitors conferred a significant survival advantage in overall survival at 1-, 3- and 5-years post-transplant (RR: 1.07, 1.1, and 1.18, respectively), and a significantly lower rate of HCC recurrence (RR: 0.67) compared with conventional CNI-based immunosuppression.

#### Summary of recommendations and expert opinion

In all HCC patients (regardless of their risk of relapse), strategies to facilitate CNI reduction should be implemented to limit CNI-related renal toxicities and the impact of CNI exposure on cancer recurrence. Steroid-free immunosuppressive schedules are preferred (Fig. 4).

### Patients with de novo malignancies

#### Definition/description of patient category

Liver transplant recipients who develop de novo malignancies any time following transplantation, including extrahepatic solid tumors, non-melanoma skin cancer, and lymphoproliferative diseases.

#### Relevant supporting evidence from the literature

A relatively large body of evidence from studies in heart, kidney, and liver transplant recipients suggests that the use of mTOR inhibitors leads to a reduced incidence of de novo malignancies after solid organ transplantation [15, 56, 57]. The incidence of de novo malignancy within 963 days post-transplant was significantly lower in patients receiving sirolimus/everolimus compared with cyclosporine/tacrolimus (0.60% vs. 1.81%; *p* < 0.001) in a retrospective analysis of 33,249 kidney transplant recipients, with the risk of developing any de novo malignancy reduced by 60% with sirolimus/everolimus immunosuppression (relative risk 0.4, 95% CI 0.24–0.64; *p* = 0.0002) [56]. A retrospective cohort study of 7217 kidney transplant recipients demonstrated a 46% significantly reduced risk of de novo malignancies in recipients treated with mTOR inhibitors compared with those never treated (*p* < 0.05) [57].

Further evidence supports the use of mTOR inhibitors in patients with de novo malignancies after liver transplantation [58–60]. In 6 liver transplantation patients who converted from CNI to everolimus due to de novo malignancies, which appeared 3–4 years post-transplantation, 83% (*n* = 5) of patients remained disease-free after a mean follow-up time of 10 ± 9 months; lung carcinoma recurred at 12-months following conversion in 1 patient [58]. From a total of 4 liver transplantation recipients who were converted to sirolimus-based immunosuppression due to the development of aggressive de novo malignancies, 3 patients who converted within 90–120 days after surgical removal of the malignancy remained tumor-free at 12–33 months post-conversion; 1 patient, who switched to sirolimus at 18 months after a mastectomy for breast cancer, died due to its recurrence [59].

In this setting, mTOR inhibitors may be used either as monotherapy or combined with reduced-exposure CNI, depending on individual risk factors (i.e., time post-transplantation, transplantation indication, age at transplantation, etc.) [15, 47, 48, 58–60].

#### Summary of recommendations and expert opinion

CNI reduction or discontinuation is recommended (Fig. 5). Given its antiproliferative properties, everolimus should be administered as soon as possible. mTOR inhibitor-based immunosuppression is warranted in all liver transplant recipients at high risk of de novo malignancies (i.e., human herpes virus-8-positive patients; patients with alcoholic cirrhosis; patients with concurrent inflammatory bowel disease; recipients of grafts from donors at risk of transmission of malignancies; Epstein Barr virus-DNA positivity after transplantation) [15].

### Acute rejection of the liver graft

Based on the available evidence and experience of the scientific board members, the current guidelines were focused more on the mitigation of immunosuppression-related adverse events than on the treatment of acute rejection of the liver graft. For the management of acute rejection episodes, the scientific board invites the use of the International Liver Transplantation Society consensus guidelines on immunosuppression in liver transplant recipients [61].

## Discussion

Although liver transplantation is well consolidated in Italy and elsewhere, a large amount of heterogeneity is apparent in the immunosuppression protocols used by different transplant centers. This is partly due to the observation that acute rejection of the liver graft does not affect graft or patient survival in adult liver transplantation, whilst focus is currently on the mitigation of immunosuppression-related adverse events [15]. As well as providing guidance to transplant physicians in the implementation of current recommendations for immunosuppressive therapy, the project undertaken in 2017 by the Italian Working Group aimed at standardizing immunosuppression regimens used in clinical practice with adult, ABO-compatible recipients. By dividing the population of liver transplant recipients into five distinct categories and by designing specific protocols for each category, the evidence- and consensus-based algorithms developed within the Italian Working Group, and presented here, may contribute to simplify, personalize, and optimize immunosuppression in clinical practice.

It is important to monitor renal function with adequate tools (i.e., eGFR) across all categories of liver transplant recipients. However, there remain a number of issues that are still controversial/unresolved including the management of proteinuria, and the need for additional evidence for the management of critically ill patients. The role and relevance of mTOR inhibitors in the prevention of HCC recurrence are; not definitively ascertained and need more scientific in-depth work. Evidence supporting the use of mTOR inhibitors in patients with HCC recurrence is also very limited.

## Conclusion

In conclusion, the choice of the immunosuppressive regimen in liver transplantation should take into account a composite of clinical variables, including primary disease, patient status at transplantation, type of surgery, early post-operative events, and the expected complications associated with long-term use of CNI, namely acute and chronic renal failure and de novo malignancies. Strategies for the prevention or limitation of CNI-related adverse events should be implemented as early as possible following transplantation, according to patient clinical status. At present, the most effective nephroprotective strategies include the reduction of CNI exposure facilitated by the early introduction of everolimus, or mycophenolate mofetil if everolimus is not indicated.

## Appendix A

### Names and affiliations of clinicians from Italian liver transplant centers who took part in the consensus panel

Alfonso Avolio, Policlinico Universitario A. Gemelli—Roma

Davide Bitetto, Azienda Ospedaliero Universitaria di Udine—Udine

Patrizia Boccagni, Università di Padova—Padova

Amedeo Carraro, Azienda Ospedaliera Universitaria Integrata—Verona

Antonino Castellaneta, Azienda Ospedaliera Consorziale Policlinico di Bari—Bari

Michele Colledan, Azienda Socio Sanitaria Territoriale Papa Giovanni XXIII—Bergamo

Nicola De Maria, Azienda Ospedaliero Universitaria Policlinico di Modena—Modena

Fabrizio Di Benedetto, Azienda Ospedaliero Universitaria Policlinico di Modena—Modena

Alfonso Galeota Lanza, Azienda Ospedaliera Antonio Cardarelli—Napoli

Ivan Gardini, Presidente dell’associazione di pazienti EpaC onlus

Nicola Guglielmo, Polo Ospedaliero San Camillo-Forlanini-Spallanzani—Roma

Federica Invernizzi, Policlinico di Milano—Milano

Andrea Laurenzi, Polo Ospedaliero San Camillo-Forlanini-Spallanzani—Roma

Gioacchino Leandro, Specialista in Gastroenterologia, Epatologia e Metodologia della ricerca

Ilaria Lenci, Policlinico Ospedaliero Universitario Tor Vergata—Roma

Dario Lorenzin, Azienda Ospedaliero Universitaria di Udine—Udine

Laura Mameli, Azienda Ospedaliera G. Brotzu—Cagliari

Tommaso Maria Manzia, Policlinico Ospedaliero Universitario Tor Vergata—Roma

Anna Mariani, Ospedale Niguarda Ca’ Granda—Milano

Gianluca Mennini, Azienda Policlinico Umberto I—Roma

Stefano Mirabella, Azienda Ospedaliera Città della Salute e della Scienza—Torino

Maria Cristina Morelli, Policlinico S. Orsola-Malpighi—Bologna

Daniele Nicolini, Ospedali Riuniti di Ancona—Ancona

Stefania Petruccelli, Azienda Ospedaliero Universitaria Policlinico di Modena—Modena

Enrico Regalia, Istituto Nazionale dei Tumori di Milano—Milano

Paolo Reggiani, Policlinico di Milano—Milano

Stefania Roselli, Azienda Ospedaliera Consorziale Policlinico di Bari—Bari

Fausto Zamboni, Azienda Ospedaliera G. Brotzu—Cagliari

## Abbreviations

ADPKD: Autosomal dominant polycystic kidney disease
AILD: Autoimmune liver disease
CCA: Cholangiocarcinoma
CI: Confidence interval
CNIs: Calcineurin inhibitors
eGFR: Estimated glomerular filtration rate
HCC: Hepatocellular carcinoma
IQR: Interquartile range
MDRD: Modification of Diet in Renal Disease
MELD: Model for End-Stage Liver Disease
metaCRC: Metastatic colorectal cancer
NET: Neuroendocrine tumors
PLD: Polycystic liver disease
PCLD: Isolated polycystic liver disease
RR: Risk ratio
SITO: Italian Society for Organ and Tissue Transplantation
