# Correction to: Immunosuppressive regimens for adult liver transplant recipients in real-life practice: consensus recommendations from an Italian Working Group

**DOI:** 10.1007/s12072-021-10178-7

**Published:** 2021-03-29

**Authors:** Umberto Cillo, Luciano De Carlis, Massimo Del Gaudio, Paolo De Simone, Stefano Fagiuoli, Francesco Lupo, Giuseppe Tisone, Riccardo Volpes, the Italian Liver Transplant Working Group, Alfonso Avolio, Davide Bitetto, Patrizia Boccagni, Amedeo Carraro, Antonino Castellaneta, Michele Colledan, Nicola De Maria, Fabrizio Di Benedetto, Alfonso Galeota Lanza, Ivan Gardini, Nicola Guglielmo, Federica Invernizzi, Andrea Laurenzi, Gioacchino Leandro, Ilaria Lenci, Dario Lorenzin, Laura Mameli, Tommaso Maria Manzia, Anna Mariani, Gianluca Mennini, Stefano Mirabella, Maria Cristina Morelli, Daniele Nicolini, Stefania Petruccelli, Enrico Regalia, Paolo Reggiani, Stefania Roselli, Fausto Zamboni

**Affiliations:** 1 Hepatobiliary and Liver Transplant Unit, University Hospital of Padua, Padua, Italy; 2 Department of General Surgery and Transplantation, Niguarda Hospital, Milan, Italy; 3 School of Medicine, University of Milano-Bicocca, Milan, Italy; 4 Department of General Surgery and Transplantation, Policlinico S. Orsola-Malpighi, Bologna, Italy; 5 Hepatobiliary Surgery and Liver Transplantation Unit, University of Pisa Medical School Hospital, Pisa, Italy; 6 Gastroenterology Hepatology and Transplantation, Papa Giovanni XXIII Hospital, Piazza OMS, 124127 Bergamo, Italy; 7 Department of General Surgery, Azienda Ospedaliera Città Della Salute E Della Scienza, Turin, Italy; 8 Department of Experimental Medicine and Surgery, Transplantation Surgery, Policlinico Tor Vergata University of Rome, Rome, Italy; 9 Mediterranean Institute for Transplantation and Advanced Specialized Therapies (ISMETT/IRCCS), Palermo, Italy

## Correction to: Hepatology International https://doi.org/10.1007/s12072-020-10091-5

In the original publication of this article, after the name of the last author, the wording *on behalf of the Italian Liver Transplant Working Group* is missing.

All the names mentioned in the Appendix A are collaborators to be indexed.

